# Supplementary figures and images for: Generation and characterization of mAb 61H9 against junctional adhesion molecule-a with potent antitumor activity
Source: PeerJ. 2024 Mar 14;12:e17088. doi: 10.7717/peerj.17088 (PMC10944630; doi:10.7717/peerj.17088)

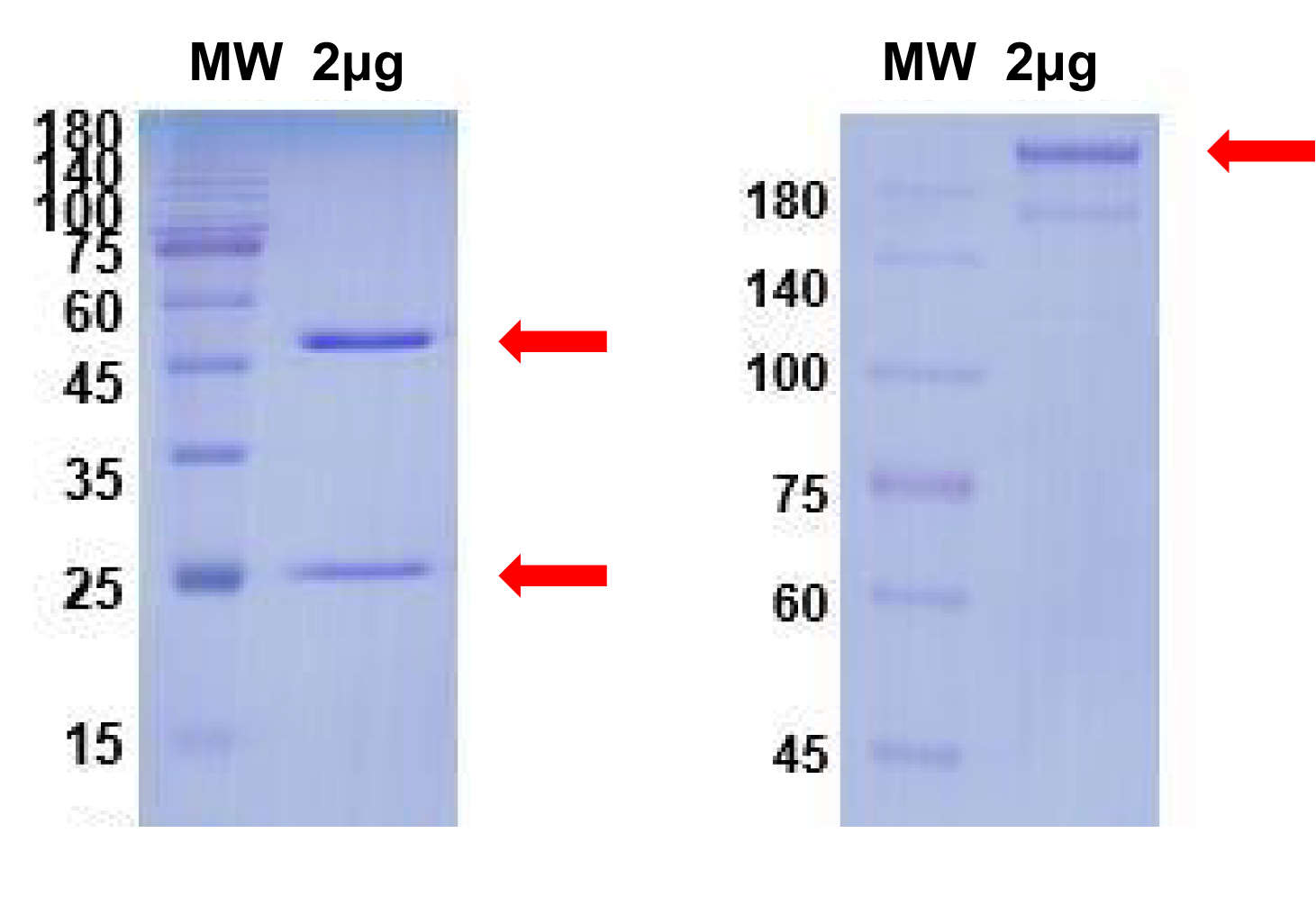

Supplement: Supplemental Information 7 — (A) Purified antibody was reductive with β-mercaptoethanol buffer. Two bands are shown around 55kD (heavy chain) and 25kD (light chain). (B) Purified antibody under non-reductive conditions . 2 μg of purified JAM-A mouse monoclonal antibody were loaded onto 10% SDS-PAGE. [file peerj-12-17088-s007.png]

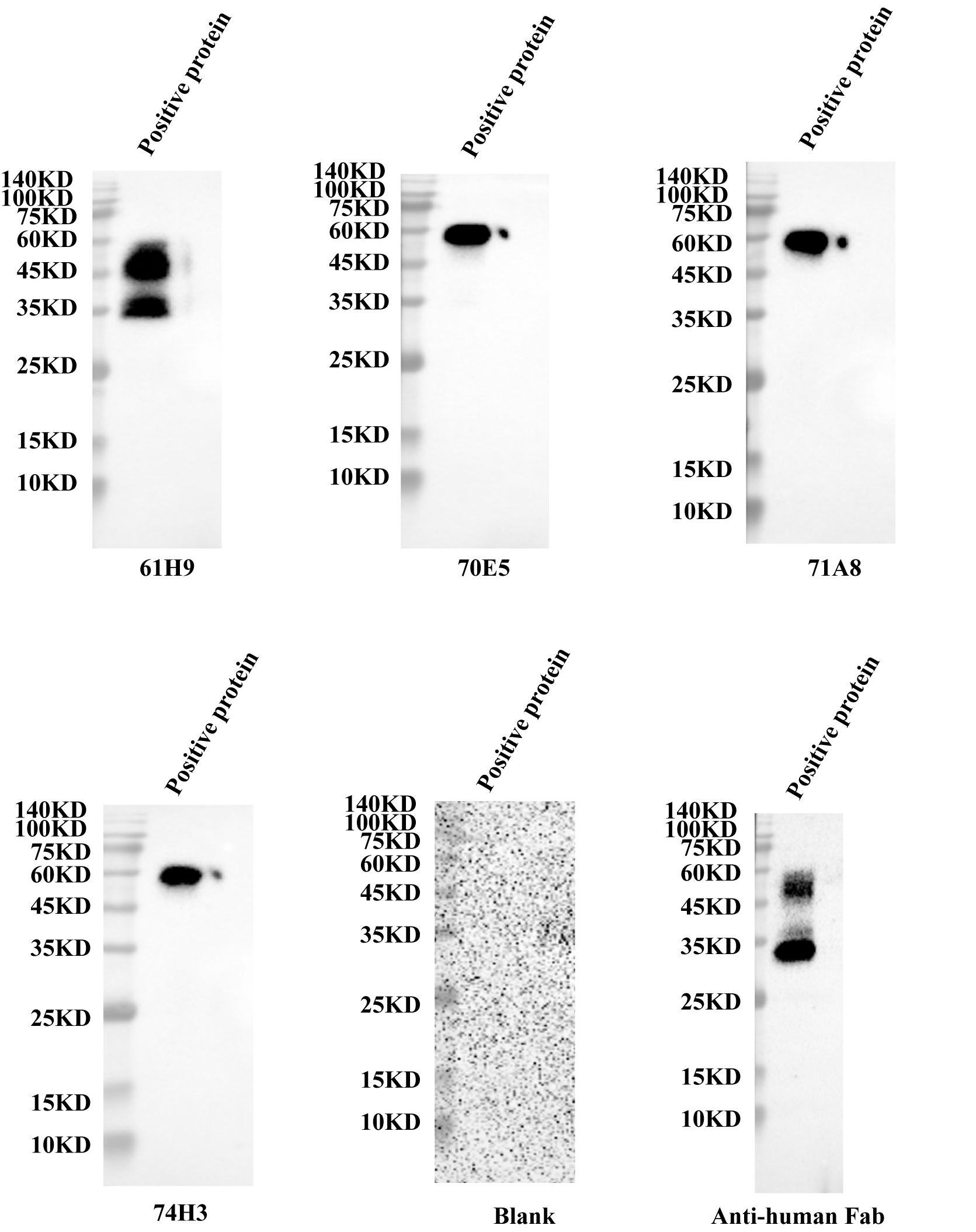

Supplement: Supplemental Information 8 — The prepared antibody to detect the endogenous protein of cells, and a positive antigen protein as the control. [file peerj-12-17088-s008.png]
